# Validation of the Internet entrepreneurial self-efficacy scale among Romanian technical students

**DOI:** 10.1371/journal.pone.0312929

**Published:** 2024-10-31

**Authors:** Beatrice Adriana Balgiu, Daniela Maricica Cotoară, Andrei Simionescu-Panait

**Affiliations:** Department of Career and Educational Training, National University of Science and Technology Politehnica Bucharest, Bucharest, Romania; Alexandru Ioan Cuza University of Iasi, Faculty of Philosophy and Social-Political Sciences, ROMANIA

## Abstract

This study’s aim is to evaluate the Romanian version of the Internet Entrepreneurial Self-Efficacy Scale (IESES), a new assessment scale for online entrepreneurial self-efficacy, and estimate its psychometric properties in a sample of engineering students (N = 644; 317 females). The scale was translated and adapted into Romanian through a forward-backward method. The factorial structure was investigated using Confirmatory Factor Analysis (CFA) and multigroup-CFA for gender invariance. The scale was associated with instruments that measure entrepreneurial intention both traditionally and in the online environment (Individual Entrepreneurial Internet Scale and Entrepreneurial Intention Questionnaire) and components from the Theory of Planned Behavior (attitudes, subjective norms and perceived behavioral control). McDonald’s omega and Cronbach’s alpha coefficients were used to assess reliability. Traditional statistics are complemented by network analysis. Results show that adequate matching items for the 3-factor model and complete gender invariance is maintained. The three factors are associated with the internet and traditional entrepreneurial intention, and the components from the theory of planned behavior (attitudes, subjective norms, and perceived behavioral control). Internal consistency is excellent because α and ω values range between 0.834 and 0.919. In addition, discriminant validity was demonstrated. The network analysis suggests the relevance of technology utilization in the self-efficacy of online businesses in the case of engineering students. Overall, findings enable us to conclude that the Romanian version of the IESES is a valid, accurate instrument that can be implemented to evaluate self-efficacy related to the successful execution of an online business.

## 1. Introduction

Recent research shows that digital transformation represents an important force impacting business innovation [1]. As far as rapid tech sales growth goes, digital entrepreneurship has become a research domain in itself because digital technologies have started to play important roles in entrepreneurial innovation and activity [2,3]. Cyber entrepreneurship (CE) [4], internet entrepreneurship (IE) [5], or digital entrepreneurship [6–8] are terms for the opportunity identification process for both innovation and business-transforming breakthrough technologies implementation. [9,10] Literature underlines the advantages of CE: stimulates innovation, models consumer values, opens up markets, reduces transaction costs, intensifies economic and social interactions through digital technologies, and increases work productivity and efficiency [11]. In addition, CE reduces initial startup costs, widens the market, and mitigates issues about founding the business. Cyber-entrepreneurs can work remotely at any time of the day [12]. CE start-ups, in general, do not need a physical space to rent [2] there being the speed and efficiency of business activities [13,14].

Students have the opportunity to start businesses in the digital sphere. A recent analysis of digital entrepreneurship perception in Romanian students shows that they are aware of the benefits of adopting ITC tools in their activities. They especially target solutions offered by social networks or cloud computers. The youngest generation sees the integration of digital technologies into business as a viable alternative [15]. The need to evaluate the key factors involved in digital entrepreneurship, such as intention, self-efficacy, perceptions and attitudes and the need for achievements in online business is a key step for universities’ efforts to develop digital entrepreneurship training programs [16].

A key in moving from intention to action in entrepreneurship is entrepreneurial self-efficacy (ESE) defined as a person’s belief about the ability to successfully fulfill various roles and responsibilities of entrepreneurship [17–19]. A series of studies demonstrate that ESE plays an important role in the entrepreneurial intention of undergraduate students [20–23]. The studies that developed instruments for ESE assessment either focused on entrepreneurial skills such as: developing new product and market opportunities, building an innovative environment, initiating investor relations, defining core purpose, coping with unexpected challenges and developing critical human resources [24], or have captured ESE within the distinct phases of the process of creating and developing a business (seeking opportunities, planning, organizing resources and implementing in relation to people and financial resources) [25,26].

However, regarding the assessment of online business self-efficacy, a measure should also capture other aspects specific to businesses in the online environment. The issue of digital skills needed in CE is not yet widely explored. CE involves developing and customizing artefacts to integrate with existing or new digital platforms, creating digital components that are part of a new digital product/service and reaching new markets and customers. It is considered that entrepreneurs who open businesses on the internet must have certain capabilities and prove their self-efficacy in relation to marketing, innovation, financial control, building an innovative environment and quick relationships with consumers [27].

Internet entrepreneurs must have the skills and abilities to create design solutions and use high-quality e-commerce systems such as websites, online platforms and social media [28]. Other authors assume that digital entrepreneurship is characterized by the harmonization of conventional entrepreneurial practices with innovative methods of creating and operating businesses in the contemporary digital age [29]. There are certain dimensions of ESE from the offline environment that can also be found online, but one of the main aspects of online business consists in the use of digital technologies and online marketing (managing ed files, the ability to install and use website applications etc.) [7,30]. However, one of the essential skills of Internet entrepreneurs is the ability to use technology to design high-quality e-commerce systems [31].

Internet entrepreneurial self-efficacy is defined as a person’s belief or confidence in launching a successful entrepreneurial venture on the Internet. This aspect comprises 5 dimensions: business operation, leadership, technology utilization, online customer service, and Internet marketing [5].

In this context, the Internet Entrepreneurial Self-Efficacy Scale (IESES) [5] was developed considering the evaluation of online entrepreneurial self-efficacy. The scale subject to validation is built to evaluate the individual’s own belief that he can successfully do business in the online environment, with an emphasis on technical skills and those related to internet marketing. The tool has as its conceptual model the theory of self-efficacy [32] derived from Social Cognitive Theory [33], adapted to the context of online entrepreneurship. The scale developed and validated in the Taiwanese context on a sample of 336 entrepreneurs is composed of 16 items included in 3 distinct factors that in the initial research explain 69.37% of the total variance of the items [5]. These items are included in the following subscales:

- *Leadership*, which looks at the capacity to lead partners and to make IE business decisions;
- *Technology utilization*, which refers to the capacity to use multimedia tools or website apps;
- *Internet marketing and e-commerce*, which is the capacity to offer high-quality services to online customers. This refers to design and e-commerce system utilization abilities (websites, online platforms, social media, etc.)

At the same time, the results supported the use of a higher-order model with 3 subdimensions. The Cronbach’s α coefficients reported by the authors are over 0.87 for the three scales and 0.94 for the total score. The correlation between factors’ IESES items ranges between 0.48 and 0.85. The scale also offers its reduced version with 3 items in which the items that obtained the highest factor loading were selected (I can make others agree with my thoughts; I can install and manipulate basic types of computer hardware to help my business; I can propose a profitable business model for electronic commerce). The scale’s short version has high correlations (r = 0.93) with the average IESES score and shows a good internal consistency (α > 0.75).

The IESES nomological validity is shown by associating IESES with internet entrepreneurship knowledge and entrepreneurial intention [5]. IESES showed correlations with scales measuring entrepreneurial education and internet entrepreneurship performance in terms of financial processes, customer satisfaction, productivity and employee satisfaction [34].

Although it was used in exploratory studies [34], there is only one adaptation of the scale in another language, namely in Spanish, in the Latin-Peruvian context on student business and industrial engineering samples [35]. IESES supports a three-dimensional structure and illustrates very good reliability (α_total score_ = 0.96). The scale is rather positively correlated to self-efficacy and negatively with academic procrastination [35].

## 2. Purpose of the study

The purpose of the current study was to adapt and clarify the psychometric properties of the Internet entrepreneurial self-efficacy scale (IESES) [5] for use with students. In order to investigate the orientation of Romanian adults over the IE, it is imperative that the structure of the measures be analyzed in the language of Romanian and that the psychometric criteria be verified.

The research background is about the paucity of Romanian instruments necessary to evaluate factors that are involved in entrepreneurial orientation and intention. To our knowledge, there is no such instrument for IE aspects in Romania and the current study fills in this gap. The IESES demonstrated good psychometric properties in its original samples [5]. The strength of the scale lies in the fact that, unlike other scales that measure entrepreneurial self-efficacy [36–38], the IESES has clearly specified within the IE the dimension of the skills to use technology internet and that related to internet marketing, skills and e-commerce. With expectations regarding the robust properties of IESES, we proposed to analyze it in the case of engineering students.

The option for the respective category of students is determined by several reasons. They tend to opt for IE more than their colleagues from other domains because of the technical and business administration knowledge they get during their studies. As studies show, IT-trained students are more capable of predicting online customers needs and preferences [39]. Second, the study of future engineering attitude and behavior is very important in a knowledge-based economy, and, implicitly, in tech-based companies [40]. Third, many authors have shown that IE self-efficacy is associated with online entrepreneurial intention [5,29,41]. This research focuses on engineering students’ entrepreneurship intentions about IE because entrepreneurship intentions can be one of the best predictors of planned behavior when starting a new business [42]. Last, our research relevance is maintained by the idea that, in Romania, the number of university alumni choosing entrepreneurship is double than that of non-graduate entrepreneurs [43].

The present study sought to examine a) the cross-cultural adaptation of the scale through forward-backward translation; b) factorial validity through CFA strategy and the reliability of the IESES. c) Also, the study integrates the perspective of the theory of planned behavior demonstrating the association of online entrepreneurial self-efficacy with entrepreneurial intention, attitudes, norms and perceived behaviors regarding online entrepreneurship; d) some practical implications.

## 3. Materials and methods

### 3.1 The scale’s cross-cultural adaptation

In the process of adapting the instrument to the Romanian cultural context, we started from the recommendations of Gudmundsson [44] prescribed in the field of adapting instruments for cross-cultural research. Thus, the English version of the scale was translated into Romanian after obtaining the consent of the main author by two independent translators familiar with the terminology of the field and fluent English speakers. For each of the items, care was taken that the equivalents in Romanian do not change the meaning of the scale intended for the answers. The two versions were compared to check for possible discords and to create a synthesis of the two versions. After refining the wording in Romanian, the working version was translated from Romanian to English by two other bilingual speakers of both languages. At this stage, both versions were analyzed and the final version was created. The pretest was carried out on a group of people from the population in which the instrument is validated [45], namely 16 students from the engineering field who were asked to comment on the understanding and clarity of the test items and instructions. They were not included in the final batch on which the research was carried out. As a result, several changes related to reframing were made that did not change the original meaning.

### 3.2. Ethical considerations

The study has been conducted in full accordance with ethical principles, including the World Medical Association from 1975 as revised in 2013. Informed consent was obtained from all participants involved in the study. This study acquired ethical approval from the relevant departamental ethics committee from the National University of Science and Technology Politehnica Bucharest (Reg. No. 3048/16.10.2023).

### 3.3. Participants and procedure

The sample sizes were calculated using the following parameters: an expected effect size of 0.30, a desired statistical power level of 0.95, a probability level of 0.05, eight latent variables, and 41 observable variables. The recommended minimum sample size is 256 respondents. We collected data from 644 students, number that surpasses Brown’s [46] minimal requirement (N = 300) for CFA.

The study is of cross-sectional type and the sample was drawn from three Romanian comprehensive technical universities: National University of Science and Technology Politehnica Bucharest (UNSTPB) and Technical University of Construction (UTCB) from Bucharest, and Polytechnic University of Timisoara (UPT) from Timișoara. The study is based on a cross-sectional design and is practically about filling in an online survey. The timeframe was 18 October to 19 December 2023. The survey link was distributed to students by the authors of the study who work with students in different disciplines. The usual time to fill in the surveys was approximately 12–14 minutes. This study’s instruments belong to broader research on technical university entrepreneurship. The survey link was secured so that each participant could fill in the survey only once. The survey was introduced by informing participants about the research purpose, procedure, and informed consent. Before completing the online survey, participants had to read the information about the purpose of the study and select the option "I agree to participate in the study". Participation was anonymous to control the social desirability effect [47]. Eligibility conditions included being over 18 years old, speaking Romanian as a native, and being enrolled in a technical university. All selected universities have entrepreneurship courses in their curricula. UNSTPB even has an Entrepreneurship and Management Engineering program. All universities often organize training programs such as the "Be an Entrepreneur 8.0" program and entrepreneurship events where top business people are invited as guest speakers.

### 3.4. Measures

1. **Internet Entrepreneurial Self-efficacy Scale–**IESES [5] contains sixteen items designed as a 7-point Likert scale, ranging from *1 –strongly disagree*, to *7 –strongly agree*. Sample items: I possess the ability to be a leader (Leadership subscale ‐ 5 items); I can install and manipulate basic types of computer hardware to help my business (Technology utilization ‐ 4 items); I can create a unique electronic commerce website (Internet marketing and e-commerce ‐ 7 items). The score for each subscale is calculated by summing up answers to subscale component items.
2. **Individual Entrepreneurial Intent Scale**–IEIS [48] measures entrepreneurial intention. The scale contains ten items. Three of them are reversed, while four of them are distracter items not bound to entrepreneurial intention. These four act as red herrings. All items are evaluated on a scale from *1 –definitely false* to *6 –definitely true*. Sample item: I intend to set up a company in the future. The scale was developed and validated on international and postgraduate student samples. It obtained Cronbach’s alpha coefficient of internal reliability between 0.84–0.91 [48] The total score resulted from summing up all item scores. In the present study it has obtained a good internal consistency of α = 0.833 [95%CI = 0.812–0.853]; ω = 0.836 [95%CI = 0.803–0.864]. CFA shows the following coefficients: χ²/df = 3.14; TLI = 0.985; CFI = 0.991; RMSEA = 0.067 [0.044–0.081]; SRMR = 0.075.
3. **Entrepreneurial Intention Questionnaire**–EIQ [49] comprises 19 items on a scale from *1 –total disagreement* to *7 –total agreement*. It captures four motivational factors that have a role in influencing entrepreneurial behavior. The scale conceived for traditional entrepreneurship was adapted for internet entrepreneurship, according to the model proposed by Tseng et al. [50]: Attitudes towards internet entrepreneurship are about the degree to which the individual self-evaluates the tendency towards IE (5 items ‐ e.g.: Being an internet entrepreneur implies more advantages than disadvantages to me); Subjective norms regarding internet entrepreneurship measures the social pressure perceived to unfold or block internet entrepreneurship activity (3 items ‐ e.g.: My friends approve of my decision to create an online firm); Perceived behavioral control over internet entrepreneurship, is defined as perceiving easiness or difficulties in becoming an entrepreneur (5 items–e.g.: I can control the creation process of a new online firm); Internet entrepreneurship intention (6 items–e.g.: I am ready to do anything to be an internet entrepreneur). In the present study, the four subscales present a good internal consistency: α scores between 0.816 and 0.955; ω between 0.819 and 0.955. The instrument illustrates a good factorial validity: χ^2^/df = 1.47; CFI = 0.995; TLI = 0.995; RMSEA = 0.039 [0.025–0.051]; SRMR = 0.061.

The last two instruments were translated from English into Romanian using forward-backward translation. Where necessary, we corrected the translations by using a re-translation process.

### 3.5. Hypotheses

The Romanian version of the IESES will show three factors, just like the original version of the instruments.

### 3.6. Sociodemographic data

A self-report questionnaire was used to collect socio-demographic information including (i) gender, (ii) age, (iii) study year, and (iv) engineering specialization.

### 3.7. Data analysis

In order to evaluate the normality of the data, the skewness and kurtosis indicators were calculated. Reliability was evaluated by the Cronbach alpha and McDonald omega coefficients whose scoring over 0.80 is considered a good result [51,52]. We used CFA for construct validity. The Diagonally Weighted Least Squares (DWLS) method is recommended for ordinal data that Likert scales have [53,54]. The model’s statistical adequacy was evaluated by focusing on goodness-of-fit indices. The relative chi-square test χ^2^/df yields acceptable results if it ranges <3 [55,56] We used a combination of indices because χ^2^ is sensitive to sample size: CFI (comparative fit index), TLI (Tucker-Lewis index), and NFI (Bentler-Bonnett Normed fit index). All of these are recommended to score ≥0.95 [55] The RMSEA (root mean squared error of approximation) and SRMR (standardized root mean square residual) values are good when they score below 0.08 [55]. Ideally, ΔCFI and the ΔRMSEA values should score lower than 0.010 to properly evaluate gender invariance [57]. We based our calculation of the scale’s composite reliability (CR) on the factor loading and of convergent validity on the Average Variance Extracted (AVE). The minimal AVE level is 0.50 [58], while for CR it is 0.70 [51] Discriminant validity was calculated by comparing AVE’s square root from each construct with its inter-construct correlation. Concurrent IESES instrument validity was calculated through Pearson correlations with IEIS and EIQ scores. Network analysis of the scale was realised to determine the central items of the scale.The following centrality indicators were used: *strength*, *closeness*, *betweenness and expected influence* [59] and shrinkage and selection operator with Extended Bayesian Information Criterion (EBICglasso). We analyzed data using SPSSv24 (IBM, New York, NY, USA) and JASP 0.17.2 (Amsterdam University, Amsterdam, The Netherlands).

## 4. Results

### 4.1. Sociodemographic characteristics of the sample

The sample is made of 644 students (M = 23.04; *SD* = 2.92), out of which 327 are male and 317 female. 40.6% are currently in their first and second years of study, while 59.6% are in their third and fourth years. Students were registered in different subfields in their universities, including medical engineering (28.7%), electrical engineering (8.3%), IT&C and Automation Sciences (30.8%), Civil Engineering (18.3%), Materials Science and Engineering (4.4%), Business Engineering and Management (9.5%).

### 4.2. Testing for common method bias

The possibility of respondent social desirability was calculated through the post-hoc evaluation procedure of the common method variance (CMV), which is Harman’s single-factor test [47]. We had an Exploratory Factorial Analysis (EFA) in which the factorial solution illustrated six distinct factors greater than 1. These make up 68% of total invariance. The first factor captures 38% of data variance and scores below the 50% recommended threshold [60]. CFA shows a weak model fit: χ^2^ = 7481.639; χ^2^/df = 10.65; CFI = 0651; TLI = 0.640; RMSEA = 0.122; SRMR = 0.102. This result indicated that the single-factor model is not acceptable and has no common method bias to measure data in our study.

### 4.3. The descriptive analysis of items and factors

We follow Kim’s [61] recommendation for analyzing data normality: for samples greater than 300 who score in skewness over 2 and in kurtosis over 7, we can use the value as a reference to declare data non-normality. Absolute values of skewness (between 0.004 and -1.289), and kurtosis (between 0.110 and 1.609) illustrate data distribution normality for the Romanian version of IESES items (Table 1).

**Table 1:** The descriptive statistics of the items.

Items	M	*SD*	Min.-Max.	Skewness	Kurtosis	ω if item deleted	Corrected item-total correlation
1.	5.276	1.425	1–7	-0.553	-0.189	0.917	0.516
2.	5.173	1.345	1–7	-0.415	-0.237	0.916	0.535
3.	5.218	1.365	1–7	-0.525	-0.247	0.916	0.546
4.	6.018	1.150	1–7	-1.289	1.609	0.918	0.421
5.	5.316	1.315	1–7	-0.586	0.110	0.917	0.505
6.	5.082	1.585	1–7	-0.490	-0.544	0.917	0.520
7.	3.975	1.890	1–7	0.062	-1.016	0.915	0.605
8.	4.104	1.842	1–7	-0.004	-0.971	0.911	0.691
9.	5.781	1.453	1–7	-1.179	0.865	0.918	0.427
10.	4.380	1.660	1–7	-0.126	-0.727	0.909	0.733
11.	3.903	1.831	1–7	0.033	-1.003	0.912	0.634
12.	4.021	1.781	1–7	0.025	-0.929	0.910	0.720
13.	4.256	1.730	1–7	-0.166	-0.801	0.910	0.734
14.	4.048	1.725	1–7	-0.038	-0.850	0.910	0.747
15.	4.127	1.696	1–7	-0.062	-0.791	0.910	0.715
16.	3.594	1.733	1–7	0.191	-0.808	0.913	0.627

*Note*: M-Mean; *SD*-Standard deviation.

Item 4 (I could have pleasant conversations with my work partners) has the highest mean value (M = 6.018; *SD* = 1.150). Item 16 (I can solve tariff problems pertaining to importing and exporting) has the lowest mean value (M = 3.594; *SD* = 1.733). Corrected item-to-total correlations for all sixteen items score over the recommended minimal value of 0.40 [62].

### 4.4. Reliability

Reliability proved to be excellent for all IESES subscales: Leadership - α = 0.834; ω = 0.837; Technology utilization - α = 0.834; ω = 0.837; Internet marketing and e-commerce *-* α = 0.834; ω = 0.837). Internal consistency of 16-item total score of IESES was excellent as well (*α* = 0.916 and ω = 0.919) (Table 2). Comparable scores were obtained for internal consistency in males (α = 0.917 [0.903–0.929]; ω = 0.919 [0.906–0.932]), and females (α = 0.915 [0.900–0.928]; ω = 0.918 [0.905–0.931]).

**Table 2:** Internal consistency, averages and standard deviations for the IESES.

Factors	M*	*SD*	α [_95%_CI]	ω [_95%_CI]	Range interitem correlation
LD	5.400	1.026	0.834[0.813–0.854]	0.837[0.817–0.857]	0.382–0.653
TU	4.735	1.369	0.818[0.795–0.839]	0.844[0.825–0.864]	0.354–0.809
IMeC	4.047	1.428	0.920[0.910–0.929]	0.921[0.912–0.930]	0.469–0.764
IESES total score	4.727	1.070	0.916[0.906–0.925]	0.919 [0.909–0.928]	

*The total score obtained for every scale was divided to the number of scale items.

*Note*: LD ‐ Leadership; TU ‐ Technology utilization; IMeC ‐ Internet marketing and e-commerce.

### 4.5. Factorial structure

The Mardia coefficient is 84.527, while the critical ratio (c.r) is 44.689, so the data qualifies as non-normal multivariate. We, therefore, applied to robust bootstrap with 2000 resamplings (95% confidence interval) to solve the non-normal aspect [63].

The first hypothetical measurement model included a single latent factor in which all 16 items were included. No limit was imposed on the intercorrelation of item errors. The obtained model was not satisfactory given the χ^2^/df, and RMSEA and SRMR scores, despite having CFI, TLI, and NFI at an acceptable level of 0.93: χ^2^ = 855.369; df = 104; χ^2^/df = 8.22; CFI = 0.938; TLI = 0.930; NFI = 0.930; RMSEA = 0.126 [_90%_CI: 0.115–0.129]; SRMR = 0.110; p <0.001.

**Fig 1:**
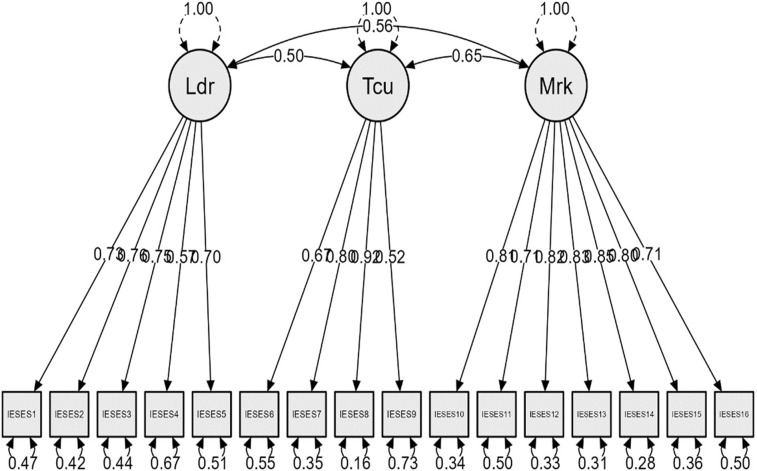
The confirmatory factor analysis of the IESES. Note: Ldr ‐ Leadership; Tcu ‐ Technology utilization; Mrk ‐ Internet marketing and e-commerce.

The three-factor model showcases higher fit coefficients than the one-factor model: χ^2^ = 227.696; df = 101; χ^2^/df = 2.25; CFI = 0.990; TLI = 0.988; NFI = 0.981; RMSEA = 0.044 [_90%_CI: 0.037–0.052]; SRMR = 0.056; p<0.001 (Table 3). Thus, we kept this one in our analysis. The item with the highest factorial loading is item number 8 (I can use multi-media hardware to help my business ‐ 0.918). Across subscales, items number 2 (I can make others agree with my thoughts ‐ 0.761; LD subscale), 8 (TU subscale) and 14 (I can propose a profitable business model for electronic commerce ‐ 0.851; IMeC subscale) stand out (Mean λ = 0.747) (Fig 1).

**Table 3:** Goodness-of-fit indices of the CFAs.

Models	χ^2^	df	χ^2^/df	CFI	TLI	NFI	RMSEA [_90%_CI]	SRMR
M1: one factor	855.369	104	8.22	0.938	0.930	0.930	0.126 [0.099–0.113]	0.110
M2: three-factors	227.696	101	2.25	0.990	0.988	0.981	0.044 [0.037–0.052]	0.056

### 4.6. The measurement invariance across gender

Gender invariance results have shown that the scale yields the same concept in both male and female groups. Scores of the ΔCFI (0.005) and ΔRMSEA (between -0.007 and -0.006), lower than recommended limits, maintain gender-specific invariance. Therefore, IESES is invariant when it comes to male and female samples (Table 4).

**Table 4:** The gender invariance for IESES.

Model Invariance	Overall Fit Indices					Comparative Fit Indices			
	χ^2^	df	χ^2^/df	CFI	RMSEA	Δχ^2^	Δdf	ΔCFI	ΔRMSEA
Configural	663.225	448	1.48	0.989	0.035 [0.029–0.041]	–	–	–	–
Metric	784.078	467	1.67	0.984	0.042 [0.037–0.047]	-120.853	-19	0.005	-0.007
Scalar	817.324	486	1.68	0.984	0.042 [0.037–0.047]	-154.099	-38	0.005	-0.007
Strict	832.529	509	1.63	0.984	0.041 [0.036–0.048]	-169.304	-61	0.005	-0.006

### 4.7. Convergent validity and discriminant validity

Average variance extracted (AVE) and CR (composite reliability) were estimated upon the factorial load (λ), and on the standard measurement error (ε) obtained in CFA. The CR (0.825–0.921) and AVE (0.497–0.627) scores are situated above minimal 0.70 [64], and 0.50 levels. The only exception is the LD subscale which has its AVE at 0.497. According to Fornell and Larcker [65], AVE < 0,5 can be accepted if CR scores over 0.60 because the construct convergent validity is adequate [66]. The Fornell-Larcker criterium [65], which compares AVE’s square root for each construct with its inter-construct correlation, was also applied to determine the scale’s discriminant validity (Table 5). Results prove that AVE’s square root is higher than correlations from its corresponding lines and columns, thus indicating the presence of discriminant validity.

**Table 5:** Convergent and discriminant validity.

Factors	CR	AVE	1	2	3
LD	0.831	0.497	**0.704** *		
TU	0.825	0.551	0.440	**0.742** *	
IMeC	0.921	0.627	0.488	0.570	**0.791** *

*Note*: LD ‐ Leadership; TU ‐ *Technology utilization*; IMeC ‐ *Internet marketing and e-commerce*.

* Square root of AVE value for each factor.

### 4.8. Criterion-related validity

We used the Wang et al. [5] method to evaluate criteria validity, namely, we chose a global item: “Overall, I feel very confident about e-commerce entrepreneurship.” The correlation between the mean IESES score and the global item’s score was significant and positive (r = 0.780; p <0.001). This shows that criteria validity is present. The correlations between the criteria and subscales were also significant and positive: Leadership (r = 0.460), Technology utilization (r = 0.452), and Internet marketing and e-commerce (r = 0.850) (all at p<0.001).

### 4.9. Concurrent validity

As expected, the results of bivariate correlations illustrate statistically significant correlations between IESES subscales and EIQ and IEIS subscales. All three IESES subscales are significantly correlated with IEIS’s entrepreneurial intention (EI) (r between 0.35 and 0.59) and from EIQ (r between 0.35 and 0.58). They were also significantly correlated to attitude regarding startup (PA, r scored between 0.35 and 0.56), to subjective norms (SN, r scored between 0.26 and 0.45), and to perceived behavior control (PBC, r scored between 0.42 and 0.69). Significant moderate correlations are present between the three IESES subscales (r between 0.44 and 0.57, all are at p <0.001) (Table 6).

**Table 6:** Intercorrelations between IESES and the other measures (IEIS and EIQ)*.

Tools	Vars.	1	2	3	4	5	6	7
IESES	1.LD	–						
	2.TU	0.440	–					
	3.IMeC	0.488	0.570	–				
IEIS	4.EI	0.401	0.352	0.595	–			
EIQ	5.PA	0.513	0.367	0.563	0.685	–		
	6.SN	0.455	0.373	0.361	0.239	0.482	–	
	7.PBC	0.480	0.430	0.700	0.702	0.698	0.313	–
	8.IEI	0.454	0.377	0.586	0.757	0.843	0.349	0.804

*Note*: LD ‐ Leadership; TU ‐ *Technology utilization*; IMeC ‐ *Internet marketing and e-commerce; EI entrepreneurial intention; PA ‐ personal attitudes*, *SN ‐ subjective norm; PBC ‐ Perceived behavioral control; IEI ‐ internet entrepreneurial intention*.

* all correlations are significant at p<0.001.

### 4.10. Network analysis

**Fig 2:**
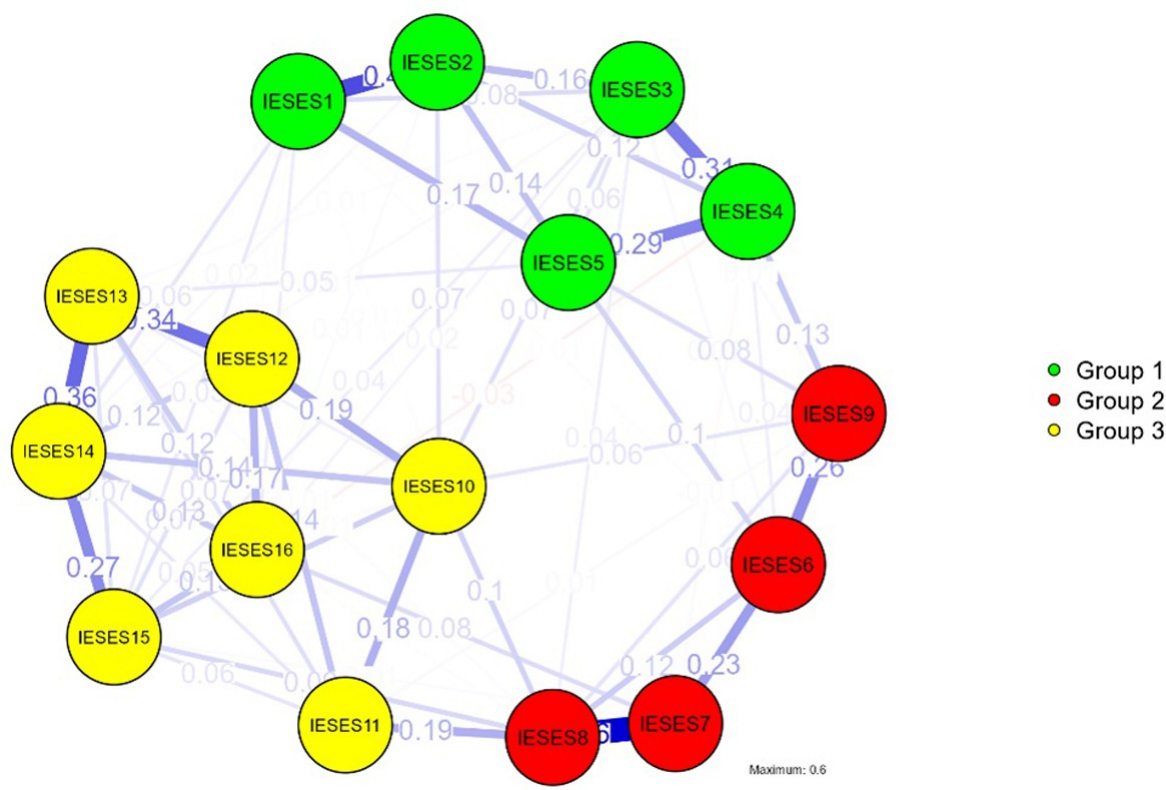
Network plots of IESES. Nodes represent items of the IESES.

A visual representation of the network of items in the IESES is shown in Fig 2 from which it can be seen that positive values were obtained between all the items of the scale. Nodes represent the items and edges represent the interaction between them. The connectivity between the nodes shows the strongest correlation between items 7 and 8 (r = 0.60), followed by that between items 1 and 2 (r = 0.42). The three obtained clusters (groups 1–3) correspond to the three factors of the scale. Nodes with high centrality index values are considered the most relevant in the network (Table 7).

**Table 7:** Centrality indicators per items.

Items	Betweenness	Closeness	Strength	Expected influence
IESES1	0.087	0.769	0.730	0.713
IESES2	0.174	0.754	0.781	0.781
IESES3	0.174	0.804	0.677	0.677
IESES4	0.522	0.805	0.861	0.746
IESES5	0.304	0.813	0.766	0.734
IESES6	0.870	0.896	0.650	0.650
IESES7	1.000	0.933	0.810	0.757
IESES8	0.826	0.918	1.000	1.000
IESES9	0.435	0.827	0.574	0.556
IESES10	0.609	1.000	0.803	0.803
IESES11	0.478	0.904	0.606	0.574
IESES12	0.609	0.902	0.862	0.862
IESES13	0.304	0.842	0.867	0.867
IESES14	0.261	0.849	0.946	0.946
IESES15	0.174	0.805	0.731	0.731
IESES16	0.000	0.748	0.629	0.567

The procedure consisted of analyzing the centrality of the items using the mentioned indicators: Betweenness (evaluates how many times a node is on the shortest path between two other nodes, identifying nodes that act as "bridges" between other nodes in the network), Closeness (quantifies how well is a node indirectly connected to other nodes), strength (indicates which node has the strongest direct connections) and expected influence (calculated to overcome the potential uncertainty of the traditional centrality measure in the case of weighted networks with both positive and negative edges). In terms of centrality indicators, items 8 (I can use multi-media hardware to help my business) and 14 (I can propose a profitable business model for electronic commerce) have the highest centrality indices, while items 9 (I have the ability to install and use website applications), 11 (I can create a unique electronic commerce website) and 16 (I can solve tariff problems pertaining to importing and exporting) have the weakest connection with the network. The network analysis indicates that the essence of internet self-efficacy for students is linked to the technological and e-commerce aspects.

## 5. Discussion

The study’s main aim is to evaluate IESES reliability and validity on Romanian samples. Results confirm research hypotheses. Thus, the construct validity highlights the presence of the three factors in our sample, similar to what Wang et al. [5] had. Although the original IESES was developed and validated on a convenient Taiwanese entrepreneurs sample, the scale has good psychometric properties in the case of Romanian version of the IESES and its sample of tech and entrepreneurship students. Calculating gender invariance proves that IESES measures the same construct for both genders. Configural, metric, scalar, and strict models showcased perfect scores. This shows that the sample’s men and women have similar interpretations regarding the content. Two items that obtained the highest factorial load and created the scale’s short version are identical to those illustrated by Wang et al. [5] as having a high factorial load: I can make others agree with my thoughts (0.761 –LD subscale); I can propose a profitable business model for electronic commerce (0.851 –IMeC subscale); the third belongs to the scale TU scale: I can use multi-media hardware to help my business (0.918).

Convergent validity was confirmed by using AVE and CR. Cronbach’s α (between 0.834 and 0.920) and McDonald’s ω (between 0.837 and 0.921) values for the three subscales prove a good IESES reliability. Results are comparable to Wang et al.’s [5] and their original study (total α = 0.94, LD = 0.87, TU = 0.92, IMeC = 0.95). The difference lies in the TU scale. The scale in the original study [5] obtained a higher α than the value of α obtained in the present study. Internal consistency coefficient scores are similar to those obtained on entrepreneurs [5] and students [35]. Criterion validity is also confirmed. Individuals with high IESES scores are most probably going to engage in entrepreneurial activities.

Concurrent validity showcased positive relations between all IESES subscales and both traditional and online entrepreneurial intentions. All items showed factor loadings above 0.35 and converged in the right direction. The result is consistent with previous studies. For instance, Wang et al. [5] show that IE self-efficacy is correlated to IE intention. Previous studies demonstrated that CE self-efficacy facilitates CE intentions even for non-IT students [2]. Chen [67] researched student samples and saw that self-efficacy affected entrepreneurial intentions in the IT field.

All IESES scales correlate to subscales that measure theory pf planned behavior components (personal attitudes, subjective norm; perceived behavioral control) [68]. According to some previous studies, self-efficacy is always correlated to concepts such as perceived behavior control and subjective norms [69]. A broader study on student samples showcases relations between entrepreneurial self-efficacy, personal attitudes, subjective norm, perceived behavioral control, and their influence on entrepreneurial intention [70]. The network analysis brings additional information showing that for students the essence of internet self-efficacy is related to the technological and e-commerce aspects, exactly the fields in which they are better, considering the knowledge and specialization they have in engineering and in the field of business administration. However, there are problems that go beyond the students, such as those related to tariff problems pertaining to importing and exporting.

*Study limitations*. Convenience sampling has a good ecological validity [71]. However, longitudinal studies are required for an instrument’s dynamic aspects. Our study gave initial proof supporting IESES validation in tech student samples. Further research is required to extend these findings by using a longitudinal design to explore causal relations between variables. In this sense, we suggest following the same cohort of students over time to assess how their entrepreneurial self-efficacy develops and how the scale performs in measuring these changes. Secondly, the study uses self-reporting scales which, despite their good psychometric properties, can lead to self-report biases. We want to deliver supplementary proof regarding IESES validation on other population samples, but also regarding the test-retest correlation for IESES in the future.

## 6. Conclusions

Our study is the first that deals with adapting and validating IESES in a Romanian sample. Results enable us to say that the Romanian version of the IESES is a consistent, high-quality instrument for evaluating student self-perception regarding their entrepreneurial abilities in IE. Implications about research and practice surface. First, a theoretical implication is that the instrument’s psychometric properties hold in our European-Latin context, which differs from the original Asian one. The instrument illustrated good structural validity in students, despite it being designed for entrepreneurs. Second, the theoretical contribution of the study consists is to provide empirical support to the model proposed by Wang et al. [5] which proposes the presence of specific skills (technology utilization, internet marketing and e-commerce) as key factors in IE. In this way, this study provides evidence of the transculturality of the online ESE model. After all, previous studies have shown that IE essential abilities are tied to e-service and technology utilization. [28,39,72,73] Moreover, studies conclude that if engineers lack managerial skills or management students are deficient in engineering knowledge, they cannot be successful in online entrepreneurship [19,74]. It can be said that the two dimensions, the use of Internet technologies and virtual marketing are sine qua non conditions for engineers who want to open online businesses. The mentioned skills play an essential role in the creation of resources for IE. It is to the credit of IESES to be the first instrument of its kind to include them. It seems that the dimension of leadership has a less important role compared to the dimension related to commerce and technology utilization [5]. However, an essential aspect of IE besides digital technology markets is the social capital that contributes to overcoming challenges and business development [8,75] Further studies on the development of instruments can capitalize on this dimension. Thus, the IE ideas hub that each class creates encourages discussion and initiative in digital entrepreneurship. Last but not least, it is important to mention that universities and business development hubs can adopt a set of instruments on their own. This can evaluate if students have the intention of starting an online business. IESES is a valid instrument that can be used for this and can help the understanding of IE’s dominant factors. In accordance with previous studies that emphasized the need to introduce entrepreneurship courses in the curriculum that have the role of developing entrepreneurial intention and interest, we consider that universities need to develop courses that can help the flourishing of abilities for kickstarting business. It is necessary for entrepreneurial education to reinvent itself not only by including IE content in their courses, but also by intensifying the presence of IE courses throughout the BA curricula. Finally, university teachers can use the passion of IT students to stimulate them towards internet entrepreneurship.

## Supporting information

S1 Appendix (DOCX)

## References

[1] Gaglio C, Kraemer-Mbula E, Lorenz E. The effects of digital transformation on innovation and productivity: Firm-level evidence of South African manufacturing micro and small enterprises. Technol Forecast Soc Change. 2022; 182: 121785. 10.1016/j.techfore.2022.121785.

[2] Chang SH, Shu Y, Wang CL, Chen MY, Ho WS. Cyber-entrepreneurship as an innovative orientation: Does positive thinking moderate the relationship between cyber-entrepreneurial self-efficacy and cyber-entrepreneurial intentions in non-IT students? Comput Hum Behav. 2020; 107(1): 105975. 10.1016/j.chb.2019.03.039.

[3] Li J, Yao M. Dynamic evolution mechanism of digital entrepreneurship ecosystem based on text sentiment computing analysis. Front Psychol. 2021; 12: 725168. doi: 10.3389/fpsyg.2021.725168 34616339 PMC8488274

[4] Tajvidi R, Tajvidi M. The growth of cyber entrepreneurship in the food industry virtual community engagement in the COVID-19 ERA. Br Food J. 2021; 123(10), 3309–3325. 10.1108/BFJ-06-2020-0559.

[5] Wang YS, Tseng TH, Wang YM, Chu CW. Development and validation of an internet entrepreneurial self-efficacy scale. Internet Res. 2020; 30(2): 653–675. 10.1108/INTR-07-2018-0294.

[6] Kollmann T. E.Entrepreneurship: The principles of founding electronic ventures. In: Zhao F., editor. Hershey: Information Technology and Business Systems Management, IGI Global, 2008. pp. 141–155, doi: 10.4018/978-1-60566-086-8.ch001

[7] Wibowo A, Narmaditya BS, Saptono A, Effendi MS, Mukhtar S, Shafiai MHM. Does digital entrepreneurship education matter for students’ digital entrepreneurial intentions? The mediating role of entrepreneurial alertness. Cogent Educ. 2023; 10(1): 1–17. doi: 10.1080/2331186X.2023.2221164

[8] Paul J, Alhassan I, Binsaif N, Singh P, Digital entrepreneurship research: A systematic review. J Bus Res. 2023; 156.113507. 10.1016/j.jbusres.2022.113507.

[9] Nambisan S. Digital entrepreneurship: toward a digital technology perspective of entrepreneurship. Entrep Theory Practice. 2017; 41(6): 1029–1055. 10.1111/etap.122.

[10] von Briel F, Recker J, Selander L, Jarvenpaa S, Hukal P, Yoo Y, et al. Researching digital entrepreneurship: current issues and suggestions for future directions. Commun Assoc Inf Syst. 10.17705/1CAIS.04833.

[11] Kraus N, Marchenko O. Innovative-digital entrepreneurship as key link of industry x.0 formation in the conditions of virtual reality. Balt J Econ Stud. 2021; 7(1): 47–56. 10.30525/2256-0742/2021-7-1-47-56.

[12] Vafaei-Zadeh A, Ganesan V, Hanifah H, Ping Teoh A, Ramayah T. Cyber-entrepreneurial intention among students in public universities: evidence from an emerging country. Educ Inf Technol. 2023; 28: 5385–5419. doi: 10.1007/s10639-022-11362-4 36373038 PMC9638269

[13] Younis H, Katsioloudes M, Al Bakri A. Digital entrepreneurship intentions of Qatar university students motivational factors identification. Digital entrepreneurship intentions. Int J E-Ent Innov. 2020; 10(1): 56–74. 10.4018/IJEEI.2020010105.

[14] Zhai Y, Yang K, Chen L, Lin H, Yu M, Jin R. Digital entrepreneurship: global maps and trends of research. J Bus Ind Mark, 2023; 38(3): 637–655. 10.1108/JBIM-05-2021-0244.

[15] Lungu AE, Georgescu MR. Students’ perceptions on digital entrepreneurship. A preliminary study. Rev Economica. 2023; 75(1): 42–49. doi: 10.56043/reveco-2023-0004

[16] Ratzinger D, Greenman A, Mosey S. The role of universities as educators in the UK Internet startup ecosystem: Research opportunities. In: Third meeting of business creation experts from business incubators and researchers: EDHEC Business. Lille. 2013.

[17] Chen CC, Greene PG, Crick A. Does entrepreneurial self-efficacy distinguish entrepreneurs from managers? J. Bus. Venturing. 1998; 13(4): 295–316. doi: 10.1016/S0883-9026(97)00029-3

[18] Barik A, Barik LB. Scale development and scale testing of student’s entrepreneur self-efficacy (SES): Preliminary psychometric test. VFAST Transactions on Education and Social Sciences. 2016; 4(1): 25–32.

[19] Primario S, Rippa P, Secundo G. Rethinking Entrepreneurial Education: The Role of Digital Technologies to Assess Entrepreneurial Self-Efficacy and Intention of STEM Students. In: IEEE Transactions on Engineering Management. 2024; 71: 2829–2842. doi: 10.1109/TEM.2022.3199709

[20] Hockerts K. Determinants of social entrepreneurial intentions. Entrep Theory Pract. 2017; 41(1): 105–130. 10.1111/etap.12171.

[21] Newman A, Obschonkab M, Schwarz S, Cohena M, Nielsen I. Entrepreneurial self-efficacy: A systematic review of the literature on its theoretical foundations, measurement, antecedents, and outcomes, and an agenda for future research, J Voc Behav. 2019; 110: 403–419. 10.1016/j.jvb.2018.05.012.

[22] Elnadi M, Gheith MH Entrepreneurial ecosystem, entrepreneurial self-efficacy, and entrepreneurial intention in higher education: Evidence from Saudi Arabia. Int. J. Manag. Educ. 2021, 19, 100458. 10.1016/j.ijme.2021.100458.

[23] Du S, Bstieler L, Yalcinkaya G. Sustainability-focused innovation in the business-to business context: antecedents and managerial implications. J Bus Res. 2022; 138(5): 117–129, doi: 10.1016/j.jbusres.2021.09.006

[24] De Noble A.F., Jung D. and Ehrlich S.B. (1999), “Entrepreneurial self-efficacy: the development and its relationship to entrepreneurial action”, Frontier for Entrepreneurship Research, available at: https://fusionmx.babson.edu/entrep/fer/papers99/I/I_C/IC.html.

[25] McGee JE, Peterson M, Mueller SL, Sequeira JM. Entrepreneurial self-efficacy: Refining the measure. Entrep Theory Pract. 2009; 33(4):965–988. doi: 10.1111/j.1540-6520.2009.00304.x

[26] Barakat S, Boddington M, Vyakarnam S. Measuring entrepreneurial self-efficacy to understand the impact of creative activities for learning innovation. Int J Manag Educ. 2014; 12: 456–468.

[27] Santos SC, Liguori EW, Garvey E. How digitalization reinvented entrepreneurial resilience during COVID-19. Technol Forecast Soc Change. 189; 2023: 122398, doi: 10.1016/j.techfore.2023.122398 36778643 PMC9899783

[28] Duffy B.E., Pruchniewska U. Gender and self-enterprise in the social media age: a digital double bind. Information, Communication & Society. 2017; 20(6): 843–859. 10.1080/1369118X.2017.1291703.

[29] Chang SH, Wang CL, Lee JC, Yu LC Who needs entrepreneurial role models? Driving forces of students’ cyber-entrepreneurial career Intention. EURASIA J Math, Sci Tech Ed. 2018; 14(7): 3083–3098.

[30] Wrobel M. Do you have what it takes to become an Internet entrepreneur? The key competencies of successful founders. In: Richter N., Jackson P. and Schildhauer T. (Eds), Entrepreneurial Innovation and Leadership, Palgrave Pivot, Cham, pp. 51–63. 2018

[31] Wang YS, Tseng TH, Wang WT, Shih YW, Chan PY. Developing and validating a mobile catering app success model. Int J Hosp Manag. 2019; 77(4): 19–30.

[32] Bandura A. Self-Efficacy: The exercise of control, W.H. Freeman and Company, New York, 1997.

[33] Bandura A. Social foundations of thought and action: A social cognitive theory. Prentice-Hall, Englewood Cliffs, New Jersey. 1986.

[34] Yeh GH, Lin HH, Wang YM, Wang YS, Lo CW. Investigating the relationships between entrepreneurial education and self-efficacy and performance in the context of internet entrepreneurship. Int J Manag Edu. 2021; 19(3): 100565. 10.1016/j.ijme.2021.100565.

[35] Torres-Miranda JS, Ccama CA, Niño Valiente JR, Turpo-Chaparro JE, Castillo-Blanco R, Mamani-Benito O. Adaptation of the internet business self-efficacy scale for Peruvian students with a commercial profile. Front. Educ. 2024; 9:1370490. doi: 10.3389/feduc.2024.1370490

[36] Forbes DP. The effects of strategic decision making on entrepreneurial self-efficacy, Entrep Theory Practice. 2005; 29(5): 599–626. 10.1111/j.1540-6520.2005.00100.x.

[37] Hmieleski KM, Baron RA. When does entrepreneurial self-efficacy enhance versus reduce firm performance? SEJ. 2008; 2(1): 57–72. 10.1002/sej.42.

[38] Schmitt A, Rosing K, Zhang SX, Leatherbee M, A dynamic model of entrepreneurial uncertainty and business opportunity identification: exploration as a mediator and entrepreneurial self-efficacy as a moderator. Entrep Theory Practice. 2018; 42(6): 835–859. 10.1177/1042258717721482.

[39] Millman C, Li Z, Matlay H, Wong WC, Entrepreneurship education and students’ Internet entrepreneurship intentions: evidence from Chinese HEIs. J Small Bus. 2010; 17(4). 569–590. doi: 10.1108/14626001011088732

[40] Barba-Sánchez V, Atienza-Sahuquillo C. Entrepreneurial intention among engineering students: The role of entrepreneurship education, European Research on Management and Business Economics. 2018; 24(1): 53–61. 10.1016/j.iedeen.2017.04.001.

[41] Guo R, Yin H, Lv X. Improvisation and university students’ entrepreneurial intention in China: The roles of entrepreneurial self-efficacy and entrepreneurial policy support. Front. Psychol. 2022; 13:930682. doi: 10.3389/fpsyg.2022.930682 36072027 PMC9441934

[42] Fellnhofer K. The power of passion in entrepreneurship education: Entrepreneurial role models encourage passion? J Entrep Educ. 2017; 20(1): 69–98. Available from: https://www.ncbi.nlm.nih.gov/pmc/articles/PMC5985942/. 29877516 PMC5985942

[43] GEM (Global Entrepreneurship Monitor) (2023). Global Entrepreneurship Monitor 2022/2023 Global Report: Adapting to a “New Normal”. London: GEM. [Cited 25 Jan 2024] Available from: https://gemconsortium.org/file/open?fileId=51147.

[44] Gudmundsson E. Guidelines for translating and adapting psychological instruments, Nordic Psychology. 2009; 61:29–45. 10.1027/1901-2276.61.2.29.

[45] Sousa VD, Rojjanasrirat W. Translation, adaptation, and validation of instruments or scales for use in cross-cultural health care research: a clear and user-friendly guideline. Journal of Evaluation in Clinical Practice. 2010; 17: 268–274. doi: 10.1111/j.1365-2753.2010.01434.x 20874835

[46] Brown TA. Confirmatory Factor Analysis for Applied Research, 2nd ed. New York, The Guilford Press, A Division of Guilford Publications, Inc. 2015.

[47] Podsakoff PM, MacKenzie SB, Lee JY, Podsakoff NP. Common method biases in behavioral research: A critical review of the literature and recommended remedies. J Appl Psychol. 2003; 88: 879–903. doi: 10.1037/0021-9010.88.5.879 14516251

[48] Thompson E. Individual entrepreneurial intent: Construct clarification and development of an internationally reliable metric. Entrep Theory Pract. 2009; 33(3): 669–694. 10.1111/j.1540-6520.2009.00321.x.

[49] Linán F, Chen YW, Development and cross-cultural application of a specific instrument to measure entrepreneurial intentions. Entrep Theory Pract. 2009; 33(3): 593–617. 10.1111/j.1540-6520.2009.00318.x.

[50] Tseng TH, Wang YM, Lin HH, Lin SJ, Wang YS, Tsai TH. Relationships between locus of control, theory of planned behavior, and cyber entrepreneurial intention: The moderating role of cyber entrepreneurship education. Int J Manag Educ, 2022; 20(3): 100682. 10.1016/j.ijme.2022.100682.

[51] Chin W.W. How to write up and report PLS analyses. In: Esposito Vinzi V., Chin W., Henseler J.,Wang H.,eds) editors. Handbook of partial least squares. Berlin: Springer, 2010, pp. 655–690.

[52] Watkins MW, The reliability of multidimensional neuropsychological measures: From alpha to omega. Clin Neuropsychol. 2017; 31(6–7): 1113–1126. doi: 10.1080/13854046.2017.1317364 28429633

[53] Mîndrilă D, Maximum Likelihood (ML) and Diagonally Weighted Least Squares (DWLS) estimation procedures: A comparison of estimation bias with ordinal and multivariate non-normal data. Int J Digital Soc. 2010; 1(1): 60–66 Available from: http://infonomics-society.org/wp-content/uploads/ijds/published-papers/volume-1-2010/Maximum-Likelihood-ML-and-Diagonally-Weighted-Least-Squares-DWLS-Estimation-Procedures-A-Comparison-of-Estimation-Bias-with-Ordinal-and-Multivariate-Non-Normal-Data.pdf.

[54] Di Stefano C, Morgan GA. Comparison of Diagonal Weighted Least Squares Robust Estimation Techniques for Ordinal Data. Struct Equ Modeling: A Multidisciplinary Journal. 2014; 21(3): 425–438. 10.1080/10705511.2014.915373.

[55] Hooper D, Coughlan J, Mullen M. Structural equation modelling: Guidelines for determining model fit. The Electronic Journal of Business Research Methods. 2008; 6: 53–60. Available from: https://www.researchgate.net/publication/254742561_Structural_Equation_Modeling_Guidelines_for_Determining_Model_Fit.

[56] Schermelleh-Engel K, Moosbrugger H, Müller H. Evaluating the fit of structural equation models: tests of significance and descriptive goodness-of-fit measures. MPR-Online. 2003; 8: 23–74. Available from: https://www.stats.ox.ac.uk/∼snijders/mpr_Schermelleh.pdf

[57] Rutkowski L, Svetina D. Measurement invariance in international surveys: categorical indicators and fit measure performance. Appl Meas Educ. 2017; 30(1):39–51. 10.1080/08957347.2016.1243540.

[58] Hair JF, Black WC, Babin BJ, Anderson RE. Multivariate data analysis. 7th ed. Essex: Pearson Education Limited, 2014.

[59] Bringmann LF, Elmer T, Epskamp S, Krause RW, Schoch D, Wichers M, Wigman J, Snippe E. What do centrality measures measure in psychological networks? J Abn Psych. 2019; 128(8): 892–903. doi: 10.1037/abn0000446 31318245

[60] Fuller CM, Simmering MJ, Atinc G, Atinc Y, Babin BJ. Common methods variance detection in business research. J Bus Res. 2016; 69(8): 3192–3198. 10.1016/j.jbusres.2015.12.008.

[61] Kim HY. Statistical notes for clinical researchers: assessing normal distribution (2) using skewness and kurtosis. Restor Dent Endod. 2013; 38(1): 52–4. doi: 10.5395/rde.2013.38.1.52 23495371 PMC3591587

[62] Wang YS. Assessment of learner satisfaction with asynchronous electronic learning systems. Inf Manag. 2003; 41(1): 75–86. 10.1016/S0378-7206(03)00028-4.

[63] Nevitt J, Hancock GR, Performance of bootstrapping approaches to model test statistics and parameter standard error estimation in structural equation modelling. Struct Equ Modeling. 2001; 8(3): 353–377. 10.1207/S15328007SEM0803_2.

[64] Bagozzi RP, Yi Y. Specification, evaluation, and interpretation of structural equation models. J Acad Mark Sci. 2012; 40(1): 8–34. 10.1007/s11747-011-0278-x.

[65] Fornell C, Larcker DF. Evaluating structural equation models with unobservable variables and measurement error. J Mark Res. 1981; 18: 39–50. 10.2307/3151312.

[66] Rönkkö M, Cho E. An updated guideline for assessing discriminant validity. Organ Res Methods. 2022; 25(1): 6–14. 10.1177/1094428120968614.

[67] Chen L. IT entrepreneurial intention among college students: An empirical study. J Inf Syst Educ. 2013; 24(3): 233–242. Available at: https://jise.org/volume24/n3/JISEv24n3p233.pdf.

[68] Ajzen I. The Theory of Planned Behavior. Organ Behav DecisProcess. 1991; 50(2): 179–211. 10.1016/0749-5978(91)90020-T.

[69] Lestari ED, Rizkalla N, Tan AT. The influence of attitude, subjective norms, self-efficacy, locus of control, and environmental support on entrepreneurial intention. J Bus Manag Rev. 2023; 4(3): 210–224. 10.47153/jbmr43.6262023.

[70] Maheshwari G, Kha KL, Investigating the relationship between educational support and entrepreneurial intention in Vietnam: The mediating role of entrepreneurial self-efficacy in the theory of planned behavior. Int J Manag Educ. 2022; 20(2): 100553, 10.1016/j.ijme.2021.100553.

[71] Song Z, Ye J, Song X, Zhang Z, Xu P, Shen H. Development and psychometric properties of work information anxiety questionnaire. Psychol Res Behav Manag. 2023; 16:4629–4646. doi: 10.2147/PRBM.S435356 38024659 PMC10644875

[72] Hasbolah H, Rosli NM, Sidek S, Abdullah FA, Daud RRR, Khadri NAM. The digital marketing practices towards SMEs performance in cyber entrepreneurship. J Inf Syst Technol Manag. 2022; 7(27): 289–299. 10.35631/JISTM.727023.

[73] Fan M, Qalati SA, Khan MAS, Shah SMM, Ramzan M, Khan RS. Effects of entrepreneurial orientation on social media adoption and SME performance: The moderating role of innovation capabilities. PLoS ONE. 2021; 16(4): e0247320. doi: 10.1371/journal.pone.0247320 33909618 PMC8081172

[74] Zhao F, Barratt-Pugh L, Suseno Y, Standen P, Redmond J. A framework for exploring digital entrepreneurship development from a social interaction perspective. J Gen Manag. 2023; 48(2): 115–126. 10.1177/03063070211044578.

[75] Wang Y, Tian Y, Nasrullah M, Zhang R. Does social capital influence farmers’ e-commerce entrepreneurship? China’s regional evidence. Electron Commer Res. 2024. 10.1007/s10660-023-09794-2. Available at: https://link.springer.com/article/10.1007/s10660-023-09794-2.

