# Supplementary material for: Validation of the Internet entrepreneurial self-efficacy scale among Romanian technical students
Source: PLoS One. 2024 Oct 31;19(10):e0312929. doi: 10.1371/journal.pone.0312929 (PMC11527208; doi:10.1371/journal.pone.0312929)
Supplement: S1 Appendix — (DOCX) [file pone.0312929.s001.docx]

**Appendix**

The Romanian language version of IESES

1. Am capacitatea de a fi lider.
2. Pot să-i fac pe alții să fie de acord cu ceea ce eu gândesc.
3. Pot găsi parteneri de lucru/ potențiali colaboratori care completează abilitățile mele.
4. Pot avea conversații plăcute cu persoanele cu care colaborez.
5. Am capacitatea de a lua decizii după deliberare.
6. Am abilități de bază în managementul fișierelor de calculator.
7. Pot instala și manipula piese hardware din comerț pentru a-mi ajuta business-ul.
8. Pot folosi hardware multimedia pentru a-mi dezvolta afacerea.
9. Am abilitatea de a instala și utiliza aplicații din mediul online.
10. Pot formula o strategie de marketing inovativă pe internet (marketing viral).
11. Pot crea un site de e-comerce original.
12. Știu cum să formulez o strategie a prețurilor pentru magazinul meu electronic.
13. Pot analiza structura costurilor pentru magazinul meu online.
14. Pot propune un model de afaceri profitabil pentru comerțul online.
15. Pot obține cu ușurință acces la resurse necesare pentru a-mi opera magazinul online.
16. Pot rezolva problemele tarifare legate de import și export.
